# Non-Fragile Robust *H*∞ Filtering of Takagi-Sugeno Fuzzy Networked Control Systems with Sensor Failures

**DOI:** 10.3390/s20010027

**Published:** 2019-12-19

**Authors:** Hao Wang, Shousheng Xie, Bin Zhou, Weixuan Wang

**Affiliations:** Aeronautical Engineering Department, Air Force Engineering University, Xi’an 710038, China

**Keywords:** networked control systems, *H*∞ filter, non-fragile filtering, Takagi-Sugeno fuzzy systems, sensor failure

## Abstract

The fault-tolerant robust non-fragile *H*∞ filtering problem for networked control systems with sensor failures is studied in this paper. The Takagi-Sugeno fuzzy model which can appropriate any nonlinear systems is employed. Based on the model, a filter which can maintain stability and *H*∞ performance level under the influence of gain perturbation of the filter and sensor failures is designed. Moreover, the gain matrix of sensor failures is converted into a dynamic interval to expand the range of allowed failures. And the sufficient condition for the existence of the desired filter is derived in terms of linear matrix inequalities (LMIs) solutions. Finally a simulation example is given to illustrate the effectiveness of the proposed method.

## 1. Introduction

Along with the continuous development of industrial technology, networked control systems (NCSs) have gradually become a new trend and attracted much attention [1,2,3]. Compared with traditional point-to-point control systems, NCSs have advantages such as high reliability, reduced weight, low cost, and ease of maintenance [4,5]. The NCSs provide the low-level processing function via intelligent unit and are more conductive to the implementation of complex control algorithms [6,7,8,9]. However, the signal of NCSs is transmitted via bus network, which brings new challenges to algorithm design, such as networked-induced time-delay, packet dropout, and packet disordering.

Due to the strong nonlinear fitting characteristics of complex nonlinear systems, the Takagi-Sugeno (T-S) fuzzy model has been widely used in the study of nonlinear systems since it was proposed [10,11,12]. In [13], a new fuzzy Lyapunov function for stability analysis which depends on both fuzzy weighting functions and their first-order derivatives is proposed, and less conservative results can be obtained by the proposed method. Additionally, the filtering problem for T-S fuzzy systems with redundant channels and noise is investigated in [14]. The T-S fuzzy model provides a method of stability analysis and performance synthesis which is based on the linear approach for nonlinear systems.

**Figure 1:**
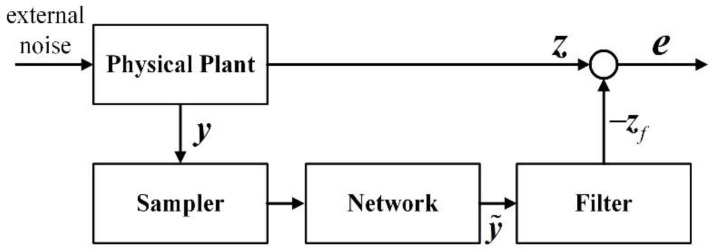
A simple diagram of filtering for networked control systems (NCSs).

In addition, the estimation of unmeasured system parameters is significantly important, since the estimated parameters can not only be used in the design of controller but also for on-line monitoring [15,16]. For this kind of problem, the current method is mainly based on filtering technology, and a simple diagram of filtering for NCSs is shown in Figure 1. Among the filters, the *H*∞ filter stands out because it has robust stability against external noise without priori knowledge of noise and precise mathematical model [17,18,19]. However, the gain perturbation of the filter is inevitable in practice, such as the error in analog-to-digital conversion and the finite word length of computer, which may affect the accuracy of the filtering results. Thus, it is desirable to design a non-fragile filter which can maintain precision with the gain perturbation of the filter.

At the same time, the parameter estimation is based on the assumption that the signal of the sensors is accurate. However, in practical applications, the sensors may fail due to electromagnetic interference or poor working environment, which will result in inaccurate filtering results or even an accident [20,21,22]. A robust *H*∞ filter is designed for a class of Markovian jump neural networks with random sensor failure in [23], but in the literature, the gain of sensor failure is only [0,1], which does not match the actual situation. In [24], the sensor failure is described as a random variable obeying the Bernoulli distribution, but the probability distribution cannot be accurately obtained. In addition, most researches only focus on a fixed mode of sensor failures, which brings limitation to the application of filter. Therefore, it has great significance and practical value to design a non-fragile filter with sensor fault tolerance to improve the safety and reliability of system.

In this paper, the robust non-fragile *H*∞ filtering problem for T-S fuzzy networked control systems with time-delay, parameter uncertainties and sensor failures is studied. The main contributions of this paper can be concluded as follows: (i) the gain perturbation of the filter is considered, so that the designed non-fragile filter has a certain range of margins for disturbance; (ii) the unknown gain matrix of sensor failures is converted into a dynamic interval, which expands the range of allowed sensor failures; (iii) the free-weighting matrix method and related linear matrix inequalities (LMIs) method are used to reduce the conservativeness of the results.

The remainder of this paper is organized as follows: Section 2 introduces the modeling method of NCSs with sensor failure and the design method of filter based on the T-S fuzzy model. The main results are presented in Section 3, including the analysis and synthesis of the filtering error system. Numerical simulation results are shown in Section 4 and finally the conclusion is presented.

## 2. Problem Formulation

Consider the NCSs with time-delay, which can be described by the T-S fuzzy model as follows:

Plant rule *i*: If $f_{1}\left(t\right)$ is $\Omega_{1}^{i}$ and ⋯ and $f_{g}\left(t\right)$ is $\Omega_{g}^{i}$, then

(1) $$\begin{array}{l} \dot{x}\left(t\right)=\left(A_{i}+\Delta A_{i}\right)x\left(t\right)+\left(B_{i}+\Delta B_{i}\right)x\left(t-\tau \left(t\right)\right)+L_{i}h(t)+\left(E_{1i}+\Delta E_{1i}\right)w(t)\\ y\left(t\right)=\left(C_{i}+\Delta C_{i}\right)x\left(t\right)+\left(D_{i}+\Delta D_{i}\right)x\left(t-\tau \left(t\right)\right)+M_{i}h(t)+\left(E_{2i}+\Delta E_{2i}\right)w(t)\\ z(t)=F_{i}x(t)+G_{i}x(t-\tau \left(t\right))+N_{i}h(t)\\ x\left(t\right)=\phi \left(t\right),t\in \left[-\tau_{m},0\right] \end{array}$$

where $\Omega_{j}^{i}\left(i=1,\cdots ,r;j=1,\cdots ,g\right)$ denotes the fuzzy set, *r* denotes the number of IF-THEN rules and $f_{j}\left(t\right)$ denotes the premise variable. $x\left(t\right)\in {\mathbb{R}}^{n}$ is state variables; $y\left(t\right)\in {\mathbb{R}}^{m}$ is measured outputs; $z(t)\in {\mathbb{R}}^{l}$ is unmeasured parameters and w(t) is the noise signal which belongs to $L_{2}\left[0,\infty \right)$. $A_{i},B_{i},C_{i},D_{i},E_{1i},E_{2i},F_{i},G_{i},L_{i},M_{i},N_{i}$ are matrices with appropriate dimension and $\Delta A_{i},\Delta B_{i},\Delta C_{i},\Delta D_{i},\Delta E_{1i},\Delta E_{2i}$ are unknown matrices which represent time-varying uncertainties. $h(t)\in {\mathbb{R}}^{p}$ is the health parameters of system, which represents the physical characteristics of each component.

**Remark 1.**  *The health parameters are considered in this paper because the performance degradation of the components in the system is inevitable during the working process. If the health parameters move away from their nominal values, the shift in other variables will be induced. In most cases, the health parameter is defined as the efficiency or capacity of each component and can be obtained by empirical formula or testing equipment. They may be treated as a set of biases and can be augmented to the system state*.

By fuzzy blending, the final augmented dynamic fuzzy model can be rewritten as

(2) $$\{\begin{array}{l} {\dot{x}}_{h}\left(t\right)=\sum_{i=1}^{r}h_{i}\left(f\left(t\right)\right)\left[\left(A_{hi}+\Delta A_{hi}\right)x_{h}\left(t\right)+\left(B_{hi}+\Delta B_{hi}\right)x_{h}\left(t-\tau \left(t\right)\right)+\left(E_{h1i}+\Delta E_{h1i}\right)w(t)\right]\\ y\left(t\right)=\sum_{i=1}^{r}h_{i}\left(f\left(t\right)\right)\left[\left(C_{hi}+\Delta C_{hi}\right)x_{h}\left(t\right)+\left(D_{hi}+\Delta D_{hi}\right)x_{h}\left(t-\tau \left(t\right)\right)+\left(E_{h2i}+\Delta E_{h2i}\right)w(t)\right]\\ z(t)=\sum_{i=1}^{r}h_{i}\left(f\left(t\right)\right)\left[F_{hi}x_{h}\left(t\right)+G_{hi}x_{h}\left(t-\tau \left(t\right)\right)\right]\\ x_{h}\left(t\right)=\left[\begin{array}{c} \phi \left(t\right)\\ 0 \end{array}\right],t\in \left[-\tau_{m},0\right] \end{array}$$

where

$$\begin{array}{l} x_{h}\left(t\right)=\left[\begin{array}{c} x\left(t\right)\\ h\left(t\right) \end{array}\right],A_{hi}=\left[\begin{array}{cc} A_{i} & L_{i}\\ 0 & I \end{array}\right],\Delta A_{hi}=\left[\begin{array}{cc} \Delta A_{i} & 0\\ 0 & 0 \end{array}\right],B_{hi}=\left[\begin{array}{cc} B_{i} & 0\\ 0 & 0 \end{array}\right],\Delta B_{hi}=\left[\begin{array}{cc} \Delta B_{i} & 0\\ 0 & 0 \end{array}\right],\\ C_{hi}=\left[\begin{array}{cc} C_{i} & M_{i} \end{array}\right],\Delta C_{hi}=\left[\begin{array}{cc} \Delta C_{i} & 0 \end{array}\right],D_{hi}=\left[\begin{array}{cc} D_{i} & 0 \end{array}\right],\Delta D_{hi}=\left[\begin{array}{cc} \Delta D_{i} & 0 \end{array}\right],E_{h1i}=\left[\begin{array}{c} E_{1i}\\ 0 \end{array}\right],\\ \Delta E_{h1i}=\left[\begin{array}{c} \Delta E_{1i}\\ 0 \end{array}\right],E_{h2i}=E_{2i},\Delta E_{h2i}=\Delta E_{2i},F_{hi}=\left[\begin{array}{cc} F_{i} & N_{i} \end{array}\right],G_{hi}=\left[\begin{array}{cc} G_{i} & 0 \end{array}\right],\\ h_{i}\left(f\left(t\right)\right)=\frac{\vartheta_{i}\left(f\left(t\right)\right)}{\sum_{i=1}^{r}\vartheta_{i}\left(f\left(t\right)\right)},\vartheta_{i}\left(f\left(t\right)\right)=\prod_{j=1}^{g}\Omega_{j}^{i}\left(f_{j}\left(t\right)\right)\;,f\left(t\right)=\left[\begin{array}{ccc} f_{1}\left(t\right) & \cdots & f_{g}\left(t\right) \end{array}\right] \end{array}$$

and $\Omega_{j}^{i}\left(f_{j}\left(t\right)\right)$ is the grade of the membership value of $f_{j}\left(t\right)$ in $\Omega_{j}^{i}$ with $\vartheta_{i}\left(f\left(t\right)\right)$ and $\sum_{i=1}^{r}\vartheta_{i}\left(f\left(t\right)\right)$. Then, there is

(3) $$h_{i}\left(f\left(t\right)\right)\geq 0,i=1,\cdots r\;\sum_{i=1}^{r}h_{i}\left(f\left(t\right)\right)=1$$

It is assumed that the uncertainties of system can be described in the following form:

(4) $$\left[\begin{array}{ccc} \Delta A_{hi} & \Delta B_{hi} & \Delta E_{h1i}\\ \Delta C_{hi} & \Delta D_{hi} & \Delta E_{h2i} \end{array}\right]=\left[\begin{array}{c} T_{1i}\\ T_{2i} \end{array}\right]K_{i}\left[\begin{array}{ccc} V_{1i} & V_{2i} & V_{3i} \end{array}\right]$$

where $T_{1i},T_{2i},V_{1i},V_{2i},V_{3i}$ are known matrices and $K_{i}$ is a time-varying unknown matrix which satisfies

(5) $$K_{i}{}^{T}K_{i}\leq I$$

Since time-delay depends heavily on variable network conditions [4], they are usually time-varying, random, and unknown. In general, the time-delay τ(t) can be divided into queuing delay $\tau_{wait}$, transmission delay $\tau_{ts}$ and reception delay $\tau_{rev}$. $\tau_{ts}$ and $\tau_{rev}$ depend on the physical characteristics of the network, and for a given networked structure, they are regular. The uncertainties of τ(t) mainly come from $\tau_{wait}$, which is affected by the network protocol. Because the load capacity of the bus is constant, there is an upper bound $\tau_{m}$ of τ(t) for a certain network protocol, and τ(t) is a random variable that is only related to the time-delay in the previous moment. The upper bound $\tau_{m}$ is constant for a certain networked structure and network protocol. It is assumed that both the sensors and actuators are time driven. The data has timestamp and are transmitted in a single-packet, and incorrect order of the data packet does not exist. Then the upper bound $\tau_{m}$ of τ(t) may be estimated; see [25] for more detail.

As a consequence, τ(t) can be modeled as a finite state Markov stochastic process on a finite set $\Omega =\left\{1,2,\cdots ,\tau_{m}\right\}$. The transition probability from τ(t)=i at time *t* to τ(t)=j (*j* ≠ *i*) at time *t*+Δ*t* is

(6) $$\mathrm{Pr}\left(\tau \left(t+\Delta t\right)=j|\tau \left(t\right)=i\right)=\left\{ \begin{array}{l} \pi_{ij}\Delta t+ο\left(\Delta t\right),i\neq j\\ 1+\pi_{ij}\Delta t+ο\left(\Delta t\right),i=j \end{array}\right.$$

where Δt>0 and $\underset{\Delta t\to 0}{\lim}\left(ο\left(\Delta t\right)/\Delta t\right)=0$. $\pi_{ij}\geq 0$ is the transition probability rates from τ(t)=i at time *t* to τ(t)=j (*j* ≠ *i*) at time *t*+Δ*t*, and there is $\sum_{j=1,j\neq i}^{\tau_{m}}\pi_{ij}=-\pi_{ii}$.

Then a more general model of sensor failures is introduced. $y^{F}\left(t\right)$ is the measured outputs with sensor failures, which has the form

(7) $$y^{F}\left(t\right)=\Theta y\left(t\right)$$

Define $\Theta =diag\left(\alpha_{1},\alpha_{2},\cdots ,\alpha_{m}\right)\left(0\leq {\underline{\alpha }}_{i}\leq \alpha_{i}\leq {\bar{\alpha }}_{i}\geq 1\right)$, and $\alpha_{i}$ is the output gain of *i*-th sensor signal. ${\bar{\alpha }}_{i}$ and ${\underline{\alpha }}_{i}$ are the upper and lower bounds of output gain, respectively, and $\alpha_{i}=1$ denotes that the *i*-th sensor is normal.

Then, the following non-fragile fuzzy filter is considered.

Filter rule *i*: If $f_{1}\left(t\right)$ is $\Omega_{1}^{i}$ and ⋯ and $f_{g}\left(t\right)$ is $\Omega_{g}^{i}$, then

(8) $$\begin{array}{l} \dot{\hat{x}}\left(t\right)=\left(A_{fi}+\Delta A_{fi}\right)\hat{x}\left(t\right)+B_{fi}y^{F}\left(t\right)\\ \hat{z}\left(t\right)=\left(L_{fi}+\Delta L_{fi}\right)\hat{x}\left(t\right) \end{array}$$

where $\hat{x}\left(t\right)\in {\mathbb{R}}^{n+p}$ is filter state and $\hat{z}\left(t\right)\in {\mathbb{R}}^{l}$ is filter output. $A_{fi},B_{fi},L_{fi}$ are the filter parameters to be determined. Similarly, $\Delta A_{fi}$ and $\Delta L_{fi}$ are variations of filter parameters, which satisfy

(9) $$\begin{array}{l} \Delta A_{fi}=T_{4i}K_{ai}V_{4i}\\ \Delta L_{fi}=T_{5i}K_{li}V_{5i} \end{array}$$

Hence, the dynamic model of non-fragile T-S fuzzy filter can be constructed as:

(10) $$\begin{array}{l} \dot{\hat{x}}\left(t\right)=\sum_{i=1}^{r}h_{i}\left(f\left(t\right)\right)\left[\left(A_{fi}+\Delta A_{fi}\right)\hat{x}\left(t\right)+B_{fi}y^{F}\left(t\right)\right]\\ \hat{z}\left(t\right)=\sum_{i=1}^{r}h_{i}\left(f\left(t\right)\right)\left[\left(L_{fi}+\Delta L_{fi}\right)\hat{x}\left(t\right)\right] \end{array}$$

Define $\xi \left(t\right)={\left[\begin{array}{cc} x_{h}^{T} & {\hat{x}}^{T}\left(t\right) \end{array}\right]}^{T}$ and $e\left(t\right)=z(t)-\hat{z}\left(t\right)$, and the filtering error system is given by

(11) $$\begin{array}{l} \dot{\xi }\left(t\right)=\sum_{i=1}^{r}h_{i}\left(f\left(t\right)\right)\left[\left({\bar{A}}_{i}+\Delta {\bar{A}}_{i}\right)\xi \left(t\right)+\left({\bar{B}}_{i}+\Delta {\bar{B}}_{i}\right)H\xi \left(t-\tau \left(t\right)\right)+\left({\bar{E}}_{i}+\Delta {\bar{E}}_{i}\right)w(t)\right]\\ e\left(t\right)=\sum_{i=1}^{r}h_{i}\left(f\left(t\right)\right)\left[\left({\bar{L}}_{i}+\Delta {\bar{L}}_{i}\right)\xi \left(t\right)+{\bar{G}}_{i}H\xi \left(t-\tau \left(t\right)\right)\right] \end{array}$$

where

$$\begin{array}{l} {\bar{A}}_{i}=\left[\begin{array}{cc} A_{hi} & 0\\ B_{fi}\Theta C_{hi} & A_{fi} \end{array}\right],\Delta {\bar{A}}_{i}=\left[\begin{array}{cc} \Delta A_{hi} & 0\\ B_{fi}\Theta \Delta C_{hi} & \Delta A_{fi} \end{array}\right],{\bar{B}}_{i}=\left[\begin{array}{c} B_{hi}\\ B_{fi}\Theta D_{hi} \end{array}\right],\Delta {\bar{B}}_{i}=\left[\begin{array}{c} \Delta B_{hi}\\ B_{fi}\Theta \Delta D_{hi} \end{array}\right],\\ {\bar{E}}_{i}=\left[\begin{array}{c} E_{h1i}\\ B_{fi}\Theta E_{h2i} \end{array}\right],\Delta {\bar{E}}_{i}=\left[\begin{array}{c} \Delta E_{h1i}\\ B_{fi}\Theta \Delta E_{h2i} \end{array}\right],{\bar{L}}_{i}={\left[\begin{array}{c} F_{hi}^{T}\\ -L_{fi}^{T} \end{array}\right]}^{T},\Delta {\bar{L}}_{i}={\left[\begin{array}{c} 0\\ -\Delta L_{fi}^{T} \end{array}\right]}^{T},{\bar{G}}_{i}={\left[\begin{array}{c} G_{hi}^{T}\\ 0 \end{array}\right]}^{T},\\ H=\left[\begin{array}{cc} I & 0 \end{array}\right]. \end{array}$$

Define the following parameters

$$\begin{array}{l} \bar{A}=\sum_{i=1}^{r}h_{i}\left(f\left(t\right)\right){\bar{A}}_{i},\Delta \bar{A}=\sum_{i=1}^{r}h_{i}\left(f\left(t\right)\right)\Delta {\bar{A}}_{i},\bar{B}=\sum_{i=1}^{r}h_{i}\left(f\left(t\right)\right){\bar{B}}_{i},\Delta \bar{B}=\sum_{i=1}^{r}h_{i}\left(f\left(t\right)\right)\Delta {\bar{B}}_{i},\\ \bar{E}=\sum_{i=1}^{r}h_{i}\left(f\left(t\right)\right){\bar{E}}_{i},\Delta \bar{E}=\sum_{i=1}^{r}h_{i}\left(f\left(t\right)\right)\Delta {\bar{E}}_{i},\bar{L}=\sum_{i=1}^{r}h_{i}\left(f\left(t\right)\right){\bar{L}}_{i},\Delta \bar{L}=\sum_{i=1}^{r}h_{i}\left(f\left(t\right)\right)\Delta {\bar{L}}_{i},\\ \bar{G}=\sum_{i=1}^{r}h_{i}\left(f\left(t\right)\right){\bar{G}}_{i}. \end{array}$$

The goal is to design a filter in the form of Equation (10) so that when there are sensor failures, the filtering error system (11) can still meet the following requirements simultaneously:

(1) The filtering error system is asymptotically stable when w(t)=0;
(2) Under the zero initial condition, the filtering error system satisfies

(12) $${\left\|e\left(t\right)\right\|}_{2}\leq \gamma {\left\|w(t)\right\|}_{2}$$

for any nonzero $w(t)\in l_{2}\left[0,\infty \right)$.

## 3. Main Results

In this section, the analysis and synthesis of the filtering error system is conducted. Before proceeding with the study, the following Lemma is needed.

**Lemma 1**  ([26])**.** D,E,F *are real matrices with appropriate dimensions, and* F *is a time-varying unknown matrix which satisfies* $F^{T}F\leq I$*. Then for a scalar* ε>0*, the following inequality*

(13) $$DFE+E^{T}F^{T}D^{T}\leq \varepsilon^{-1}DD^{T}+\varepsilon E^{T}E$$

*always holds.*

**Theorem 1.**  *If there exist positive matrices* P>0,Q>0,R>0 *and* W *such that the following inequality holds:*

(14) $$\tilde{\Gamma }=[\begin{array}{cccccc} \Phi & P\left(\bar{B}+\Delta \bar{B}\right)+H^{T}W & P\left(\bar{E}+\Delta \bar{E}\right) & \tau_{m}H^{T}W & \tau_{m}{\left(\bar{A}+\Delta \bar{A}\right)}^{T}H^{T}R & {\left(\bar{L}+\Delta \bar{L}\right)}^{T}\\ {}* & -Q & 0 & 0 & \tau_{m}{\left(\bar{B}+\Delta \bar{B}\right)}^{T}H^{T}R & {\bar{G}}^{T}\\ {}* & * & -\gamma^{2}I & 0 & \tau_{m}{\left(\bar{E}+\Delta \bar{E}\right)}^{T}H^{T}R & 0\\ {}* & * & * & -\tau_{m}R & 0 & 0\\ {}* & * & * & * & -\tau_{m}R & 0\\ {}* & * & * & * & * & -I \end{array}]<0$$

*where*

$$\Phi =P\left(\bar{A}+\Delta \bar{A}\right)+{\left(\bar{A}+\Delta \bar{A}\right)}^{T}P+H^{T}\left(Q-W-W^{T}\right)H$$

*then the filtering error system (11) is asymptotically stable and the prescribed H∞ performance* γ *is guaranteed under the zero initial condition.*

**Proof.**  Select a Lyapunov function as

(15) $$V\left(t\right)=V_{1}\left(t\right)+V_{2}\left(t\right)+V_{3}\left(t\right)$$

where

$$\begin{array}{l} V_{1}\left(t\right)=\xi^{T}\left(t\right)P\xi \left(t\right)\\ V_{2}\left(t\right)=\int_{t-\tau \left(t\right)}^{t}\xi^{T}\left(\alpha \right)H^{T}QH\xi \left(\alpha \right)d\alpha \\ V_{3}\left(t\right)=\int_{-\tau_{m}}^{0}\int_{t+\beta }^{t}{\dot{\xi }}^{T}\left(\alpha \right)H^{T}RH\dot{\xi }\left(\alpha \right)d\alpha d\beta \end{array}$$

When w(t)=0, the derivation of V(t) is

(16) $$\begin{array}{l} {\dot{V}}_{1}\left(t\right)=2\xi^{T}\left(t\right)P\left[\left(\bar{A}+\Delta \bar{A}\right)\xi \left(t\right)+\left(\bar{B}+\Delta \bar{B}\right)H\xi \left(t-\tau \left(t\right)\right)\right]\\ {\dot{V}}_{2}\left(t\right)\leq \xi^{T}\left(t\right)H^{T}QH\xi \left(t\right)-\xi^{T}\left(t-\tau \left(t\right)\right)H^{T}QH\xi \left(t-\tau \left(t\right)\right)\\ {\dot{V}}_{3}\left(t\right)\leq \tau_{m}{\dot{\xi }}^{T}\left(t\right)H^{T}RH\dot{\xi }\left(t\right)-\int_{t-\tau \left(t\right)}^{t}{\dot{\xi }}^{T}\left(\alpha \right)H^{T}RH\dot{\xi }\left(\alpha \right)d\alpha \end{array}$$

It is obvious that there is

(17) $$\xi \left(t-\tau \left(t\right)\right)=\xi \left(t\right)-\int_{t-\tau \left(t\right)}^{t}\dot{\xi }\left(\alpha \right)d\alpha$$

Then, $\dot{V}\left(t\right)$ can be rewritten as

(18) $$\begin{array}{l} \dot{V}\left(t\right)\leq 2\xi^{T}\left(t\right)P\left[\left(\bar{A}+\Delta \bar{A}\right)\xi \left(t\right)+\left(\bar{B}+\Delta \bar{B}\right)H\xi \left(t-\tau \left(t\right)\right)\right]+\xi^{T}\left(t\right)H^{T}QH\xi \left(t\right)\\ -\xi^{T}\left(t-\tau \left(t\right)\right)H^{T}QH\xi \left(t-\tau \left(t\right)\right)+\tau_{m}{\dot{\xi }}^{T}\left(t\right)H^{T}RH\dot{\xi }\left(t\right)-\int_{t-\tau \left(t\right)}^{t}{\dot{\xi }}^{T}\left(\alpha \right)H^{T}RH\dot{\xi }\left(\alpha \right)d\alpha \\ +2\xi^{T}\left(t\right)H^{T}W\left(H\xi^{T}\left(t-\tau \left(t\right)\right)-H\xi \left(t\right)+\int_{t-\tau \left(t\right)}^{t}H\dot{\xi }\left(\alpha \right)d\alpha \right)\\ \leq \frac{1}{\tau \left(t\right)}\int_{t-\tau \left(t\right)}^{t}{\left[\begin{array}{c} \xi \left(t\right)\\ H\xi \left(t-\tau \left(t\right)\right)\\ H\dot{\xi }\left(\alpha \right) \end{array}\right]}^{T}{\tilde{\Gamma }}_{0}\left[\begin{array}{c} \xi \left(t\right)\\ H\xi \left(t-\tau \left(t\right)\right)\\ H\dot{\xi }\left(\alpha \right) \end{array}\right]d\alpha \end{array}$$

where

$${\tilde{\Gamma }}_{0}=\left[\begin{array}{ccc} \Phi & P\left(\bar{B}+\Delta \bar{B}\right)+H^{T}W & \tau_{m}H^{T}W\\ {}* & -Q & 0\\ {}* & * & -\tau_{m}R \end{array}\right]+\tau_{m}\left[\begin{array}{c} {\left(\bar{A}+\Delta \bar{A}\right)}^{T}\\ {\left(\bar{B}+\Delta \bar{B}\right)}^{T}\\ 0 \end{array}\right]H^{T}RH{\left[\begin{array}{c} {\left(\bar{A}+\Delta \bar{A}\right)}^{T}\\ {\left(\bar{B}+\Delta \bar{B}\right)}^{T}\\ 0 \end{array}\right]}^{T}$$

By the Schur complement, if inequality (14) holds, there is ${\tilde{\Gamma }}_{0}<0$. So it can be obtained that there is $\dot{V}\left(t\right)<0$ and the filtering error system (11) is asymptotically stable when w(t)=0.

Secondly, a new function is defined

$$J=\int_{0}^{T}\left[e^{T}\left(t\right)e\left(t\right)-\gamma^{2}w^{T}(t)w(t)\right]dt$$

where T>0. So under the zero initial condition, there is

(19) $$\begin{array}{l} J=\int_{0}^{T}\left[e^{T}\left(t\right)e\left(t\right)-\gamma^{2}w^{T}\left(t\right)w\left(t\right)+\dot{V}\left(t\right)\right]dt-V\left(T\right)\\ \;\leq \int_{0}^{T}\left[e^{T}\left(t\right)e\left(t\right)-\gamma^{2}w^{T}\left(t\right)w\left(t\right)+\dot{V}\left(t\right)\right]dt \end{array}$$

Similarly, referring to the derivation of Equation (18), there is

(20) $$J\leq \int_{0}^{T}\frac{1}{\tau \left(t\right)}\int_{t-\tau \left(t\right)}^{t}{\left[\begin{array}{c} \xi \left(t\right)\\ H\xi \left(t-\tau \left(t\right)\right)\\ w\left(t\right)\\ H\dot{\xi }\left(\alpha \right) \end{array}\right]}^{T}{\tilde{\Gamma }}_{1}\left[\begin{array}{c} \xi \left(t\right)\\ H\xi \left(t-\tau \left(t\right)\right)\\ w\left(t\right)\\ H\dot{\xi }\left(\alpha \right) \end{array}\right]dtdu$$

where

$$\begin{array}{ll} {\tilde{\Gamma }}_{1}= & \left[\begin{array}{cccc} \Phi +{\left(\bar{L}+\Delta \bar{L}\right)}^{T}\left(\bar{L}+\Delta \bar{L}\right) & P\left(\bar{B}+\Delta \bar{B}\right)+H^{T}W+{\left(\bar{L}+\Delta \bar{L}\right)}^{T}\bar{G} & P\left(\bar{E}+\Delta \bar{E}\right) & \tau_{m}H^{T}W\\ {}* & -Q+{\bar{G}}^{T}\bar{G} & 0 & 0\\ {}* & * & -\gamma^{2}I & 0\\ {}* & * & * & -\tau_{m}R \end{array}\right]\\ & +\tau_{m}\left[\begin{array}{c} {\left(\bar{A}+\Delta \bar{A}\right)}^{T}\\ {\left(\bar{B}+\Delta \bar{B}\right)}^{T}\\ {\left(\bar{E}+\Delta \bar{E}\right)}^{T}\\ 0 \end{array}\right]H^{T}R{\left[\begin{array}{c} {\left(\bar{A}+\Delta \bar{A}\right)}^{T}\\ {\left(\bar{B}+\Delta \bar{B}\right)}^{T}\\ {\left(\bar{E}+\Delta \bar{E}\right)}^{T}\\ 0 \end{array}\right]}^{T} \end{array}$$

By the Schur complement, it follows that

$${\tilde{\Gamma }}_{1}<0\Leftrightarrow \tilde{\Gamma }<0$$

If the inequality $\tilde{\Gamma }<0$ holds, it can be obtained that there is J<0, which implies that Equation (12) holds for any nonzero $w(t)\in l_{2}\left[0,\infty \right)$. The proof is completed. □

**Theorem 2.**  *For a given constant scalar* γ>0 *, the filtering error system (11) is asymptotically stable with a H∞ performance level* γ *if there exist positive definite matrices* R>0,Q>0,S>0,X>0 *, matrices* $W,Z_{1i},Z_{2i},Z_{3i},\Lambda_{i}=\Lambda_{j}^{T}\left(1\leq i\leq r\right),\rho_{ij}\left(1\leq i\leq j\leq r\right)$ *and scalars* $\varepsilon_{1}>0,\varepsilon_{2}>0,\varepsilon_{3}>0,\mu_{1ij}\left(1\leq i\leq j\leq r\right),\mu_{2i},\mu_{3i},\mu_{4i}$ *such that the following inequalities hold:*

(21) $$\left[\begin{array}{cccc} \Lambda_{1} & \rho_{12} & \cdots & \rho_{1r}\\ {}* & \Lambda_{2} & \cdots & \rho_{2r}\\ \vdots & \vdots & \ddots & \vdots \\ {}* & * & \cdots & \Lambda_{r} \end{array}\right]<0$$

(22) $$\begin{array}{l} \;\left[\begin{array}{ccccc} \Gamma_{ii}-\Lambda_{i}+\mu_{1ii}\Xi_{1i}^{T}\Xi_{1i}+\mu_{2i}\Xi_{2i}^{T}\Xi_{2i}+\mu_{3i}\Xi_{3i}^{T}\Xi_{3i}+\mu_{4i}\Xi_{4i}^{T}\Xi_{4i} & \Psi_{1ii} & \Psi_{2i} & \Psi_{3i} & \Psi_{4i}\\ {}* & -\mu_{1ii} & 0 & 0 & 0\\ {}* & * & -\mu_{2i} & 0 & 0\\ {}* & * & * & -\mu_{3i} & 0\\ {}* & * & * & * & -\mu_{4i} \end{array}\right]<0\\ \left(1\leq i\leq r\right) \end{array}$$

(23) $$\begin{array}{l} \left[\begin{array}{ccccccccc} \Pi_{ij} & \Psi_{1ij} & \Psi_{1ji} & \Psi_{2i} & \Psi_{2j} & \Psi_{3i} & \Psi_{3j} & \Psi_{4i} & \Psi_{4j}\\ {}* & -\mu_{1ij} & 0 & 0 & 0 & 0 & 0 & 0 & 0\\ {}* & * & -\mu_{1ji} & 0 & 0 & 0 & 0 & 0 & 0\\ {}* & * & * & -\mu_{2i} & 0 & 0 & 0 & 0 & 0\\ {}* & * & * & * & -\mu_{2j} & 0 & 0 & 0 & 0\\ {}* & * & * & * & * & -\mu_{3i} & 0 & 0 & 0\\ {}* & * & * & * & * & * & -\mu_{3j} & 0 & 0\\ {}* & * & * & * & * & * & * & -\mu_{4i} & 0\\ {}* & * & * & * & * & * & * & * & -\mu_{4j} \end{array}\right]<0\\ \left(1\leq i\leq j\leq r\right) \end{array}$$

*where*

$$\begin{array}{ll} \Pi_{ij}= & \Gamma_{ij}+\Gamma_{ji}-\rho_{ij}-\rho_{ij}{}^{T}+\mu_{1ij}\Xi_{1i}^{T}\Xi_{1i}+\mu_{1ji}\Xi_{1j}^{T}\Xi_{1j}+\mu_{2i}\Xi_{2i}^{T}\Xi_{2i}+\mu_{2j}\Xi_{2j}^{T}\Xi_{2j}\\ & +\mu_{3i}\Xi_{3i}^{T}\Xi_{3i}+\mu_{3j}\Xi_{3j}^{T}\Xi_{3j}+\mu_{4i}\Xi_{4i}^{T}\Xi_{4i}+\mu_{4j}\Xi_{4j}^{T}\Xi_{4j}, \end{array}$$

$$\Psi_{1ij}={\left[\begin{array}{ccccccccccccc} T_{1i}^{T}S^{T} & T_{2i}^{T}\Theta_{0}^{T}Z_{2j}^{T} & 0 & 0 & 0 & \tau_{m}T_{1i}^{T}R^{T} & 0 & 0 & 0 & 0 & 0 & 0 & 0 \end{array}\right]}^{T},$$

$$\Xi_{1i}=\left[\begin{array}{ccccccccccccc} V_{1i} & 0 & V_{2i} & V_{3i} & 0 & 0 & 0 & 0 & 0 & 0 & 0 & 0 & 0 \end{array}\right],$$

$$\Psi_{2i}={\left[\begin{array}{ccccccccccccc} 0 & 0 & 0 & 0 & 0 & 0 & 0 & 0 & 0 & 0 & T_{2i}^{T}{ϒ}_{2}^{T} & T_{2i}^{T}{ϒ}_{2}^{T} & T_{2i}^{T}{ϒ}_{2}^{T} \end{array}\right]}^{T},$$

$$\Xi_{2i}=\left[\begin{array}{ccccccccccccc} V_{1i} & 0 & V_{2i} & V_{3i} & 0 & 0 & 0 & 0 & 0 & 0 & 0 & 0 & 0 \end{array}\right],$$

$$\Psi_{3i}=\left[\begin{array}{ccccccccccccc} 0 & T_{4i}^{T}X^{T} & 0 & 0 & 0 & 0 & 0 & 0 & 0 & 0 & 0 & 0 & 0 \end{array}\right],$$

$$\Xi_{3i}=\left[\begin{array}{ccccccccccccc} 0 & V_{4i} & 0 & 0 & 0 & 0 & 0 & 0 & 0 & 0 & 0 & 0 & 0 \end{array}\right],$$

$$\Psi_{4i}=\left[\begin{array}{ccccccccccccc} 0 & 0 & 0 & 0 & 0 & 0 & -T_{5i}^{T} & 0 & 0 & 0 & 0 & 0 & 0 \end{array}\right],$$

$$\Xi_{4i}=\left[\begin{array}{ccccccccccccc} 0 & V_{5i} & 0 & 0 & 0 & 0 & 0 & 0 & 0 & 0 & 0 & 0 & 0 \end{array}\right],$$

$$\Gamma_{ij}=\left[\begin{array}{ccccccc} \Gamma_{0ij} & \varsigma_{0} & \varsigma_{1} & \varsigma_{1} & \varsigma_{2}^{T} & \varsigma_{3}^{T} & \varsigma_{4}^{T}\\ {}* & -\varepsilon_{1}I & 0 & 0 & 0 & 0 & 0\\ {}* & * & -\varepsilon_{2}I & 0 & 0 & 0 & 0\\ {}* & * & * & -\varepsilon_{3}I & 0 & 0 & 0\\ {}* & * & * & * & -\varepsilon_{1}{}^{-1}I & 0 & 0\\ {}* & * & * & * & * & -\varepsilon_{2}{}^{-1}I & 0\\ {}* & * & * & * & * & * & -\varepsilon_{3}{}^{-1}I \end{array}\right],$$

$$\Gamma_{0ij}=\left[\begin{array}{ccccccc} SA_{hi}+A_{hi}^{T}S+Q-W-W^{T} & C_{hi}{}^{T}\Theta_{0}{}^{T}Z_{2j}^{T} & SB_{hi}+W & SE_{h1i} & \tau_{m}W & \tau_{m}A_{hi}^{T}R & F_{hi}^{T}\\ {}* & Z_{1i}+Z_{1i}^{T} & Z_{2j}\Theta_{0}D_{hi} & Z_{2j}\Theta_{0}E_{h2i} & 0 & 0 & -Z_{3i}^{T}\\ {}* & * & -Q & 0 & 0 & \tau_{m}B_{hi}^{T}R & 0\\ {}* & * & * & -\gamma^{2}I & 0 & \tau_{m}E_{h1i}^{T}R & 0\\ {}* & * & * & * & -\tau_{m}R & 0 & 0\\ {}* & * & * & * & * & -\tau_{m}R & 0\\ {}* & * & * & * & * & * & -I \end{array}\right],$$

$$\varsigma_{0}={\left[\begin{array}{ccccccc} 0 & Z_{2i}{ϒ}_{1} & 0 & 0 & 0 & 0 & 0 \end{array}\right]}^{T},\varsigma_{1}={\left[\begin{array}{ccccccc} 0 & {ϒ}_{1}{}^{T}Z_{2i}^{T} & 0 & 0 & 0 & 0 & 0 \end{array}\right]}^{T},$$

$$\varsigma_{2}=\left[\begin{array}{ccccccc} {ϒ}_{2}C_{hi} & 0 & 0 & 0 & 0 & 0 & 0 \end{array}\right],\varsigma_{3}=\left[\begin{array}{ccccccc} 0 & 0 & {ϒ}_{2}D_{hi} & 0 & 0 & 0 & 0 \end{array}\right],$$

$$\varsigma_{4}=\left[\begin{array}{ccccccc} 0 & 0 & 0 & {ϒ}_{2}E_{h2i} & 0 & 0 & 0 \end{array}\right].$$

Moreover, the parameters of fuzzy *H*∞ filter can be solved by

$$A_{fi}=X^{-1}Z_{1i},B_{fi}=X^{-1}Z_{2i},L_{fi}=Z_{3i}$$

**Proof.**  According to [27], $\tilde{\Gamma }$ can be rewritten as

(24) $$\tilde{\Gamma }=\left[\begin{array}{ccccccc} S{\tilde{A}}_{hi}+{\tilde{A}}_{hi}^{T}S+Q-W-W^{T} & {\tilde{C}}_{hi}{}^{T}\Theta^{T}Z_{2j}^{T} & S{\tilde{B}}_{hi}+W & S{\tilde{E}}_{h1i} & \tau_{m}W & \tau_{m}{\tilde{A}}_{hi}^{T}R & F_{hi}^{T}\\ {}* & X{\tilde{A}}_{fi}+{\tilde{A}}_{fi}{}^{T}X & Z_{2j}\Theta {\tilde{D}}_{hi} & Z_{2j}\Theta {\tilde{E}}_{h2i} & 0 & 0 & -{\tilde{L}}_{fi}^{T}\\ {}* & * & -Q & 0 & 0 & \tau_{m}{\tilde{B}}_{hi}^{T}R & 0\\ {}* & * & * & -\gamma^{2}I & 0 & \tau_{m}{\tilde{E}}_{h1i}^{T}R & 0\\ {}* & * & * & * & -\tau_{m}R & 0 & 0\\ {}* & * & * & * & * & -\tau_{m}R & 0\\ {}* & * & * & * & * & * & -I \end{array}\right]$$

where

$$\begin{array}{l} {\tilde{A}}_{hi}=A_{hi}+\Delta A_{hi},{\tilde{B}}_{hi}=B_{hi}+\Delta B_{hi},{\tilde{C}}_{hi}=C_{hi}+\Delta C_{hi},{\tilde{D}}_{hi}={\tilde{D}}_{hi}+{\tilde{D}}_{hi},\\ {\tilde{E}}_{h1i}=E_{h1i}+\Delta E_{h1i},{\tilde{E}}_{h2i}=E_{h2i}+\Delta E_{h2i},{\tilde{A}}_{fi}=A_{fi}+\Delta A_{fi},{\tilde{L}}_{fi}=L_{fi}+\Delta L_{fi} \end{array}$$

The gain matrix of sensor signal $\Theta =diag\left(\alpha_{1},\alpha_{2},\cdots ,\alpha_{m}\right)$ is an unknown matrix for various fault modes, which makes the design of the filter complex. However, since $\alpha_{i}$ is bounded, Θ can be transformed into a class of dynamic interval matrices.

Let $a_{i}=\frac{1}{2}\left({\bar{\alpha }}_{i}+{\underline{\alpha }}_{i}\right)$ and $b_{i}=\frac{1}{2}\left({\bar{\alpha }}_{i}-{\underline{\alpha }}_{i}\right)$, and the output gain can be rewritten as $\alpha_{i}=a_{i}+\varepsilon_{i}b_{i}\left(\left|\varepsilon_{i}\right|\leq 1\right)$. So, we define

(25) $$\Theta_{\max}={\left[diag\left({\bar{\alpha }}_{1},\cdots ,{\bar{\alpha }}_{m}\right)\right]}_{m\times m},\Theta_{\min}={\left[diag\left({\underline{\alpha }}_{1},\cdots ,{\underline{\alpha }}_{m}\right)\right]}_{m\times m}$$

Then, the dynamic interval matrices of Θ is

(26) $$\left[\Theta_{\min},\Theta_{\max}\right]=\left\{\bar{\Theta }={\left[diag\left(\alpha_{i}\right)\right]}_{m\times m}|{\underline{\alpha }}_{i}\leq \alpha_{i}\leq {\bar{\alpha }}_{i}\right\}$$

Similarly, we can define $\Theta_{0}=\frac{1}{2}\left(\Theta_{\max}+\Theta_{\min}\right),\Theta_{1}=\frac{1}{2}\left(\Theta_{\max}-\Theta_{\min}\right)={\left\{l_{ij}\right\}}_{r\times r}$, and the gain matrix can be rewritten as

(27) $$\Theta =\Theta_{0}+{ϒ}_{1}\Delta {ϒ}_{2}$$

where

$$\begin{array}{l} {ϒ}_{1}={\left[\sqrt{l_{11}}e_{1},\cdots ,\sqrt{l_{1n}}e_{1},\cdots ,\sqrt{l_{n1}}e_{n},\cdots ,\sqrt{l_{nn}}e_{n}\right]}_{m\times m^{2}},\\ {ϒ}_{2}={\left[\sqrt{l_{11}}e_{1},\cdots ,\sqrt{l_{1n}}e_{n},\cdots ,\sqrt{l_{n1}}e_{1},\cdots ,\sqrt{l_{nn}}e_{n}\right]}_{m\times m^{2}}^{T},\\ \Delta =diag{\left\{\varepsilon_{11},\cdots ,\varepsilon_{1n},\cdots ,\varepsilon_{n1},\cdots ,\varepsilon_{nn}\right\}}_{m^{2}\times m^{2}} \end{array}$$

in which $e_{i}$ is the *i*-th column of identity matrix $I_{m\times m}$. Additionally, it is obvious that there is $\Delta^{T}\Delta \leq I$.

Substituting Equation (27) into Equation (24), $\tilde{\Gamma }$ can be decomposed into

(28) $$\tilde{\Gamma }={\tilde{\Gamma }}_{2}+{\tilde{\Gamma }}_{3}$$

where

$${\tilde{\Gamma }}_{2}=\left[\begin{array}{ccccccc} S{\tilde{A}}_{hi}+{\tilde{A}}_{hi}^{T}S+Q-W-W^{T} & {\tilde{C}}_{hi}{}^{T}\Theta_{0}{}^{T}Z_{2j}^{T} & S{\tilde{B}}_{hi}+W & S{\tilde{E}}_{h1i} & \tau_{m}W & \tau_{m}{\tilde{A}}_{hi}^{T}R & F_{hi}^{T}\\ {}* & X{\tilde{A}}_{fi}+{\tilde{A}}_{fi}{}^{T}X & Z_{2j}\Theta_{0}{\tilde{D}}_{hi} & Z_{2j}\Theta_{0}{\tilde{E}}_{h2i} & 0 & 0 & -{\tilde{L}}_{fi}^{T}\\ {}* & * & -Q & 0 & 0 & \tau_{m}{\tilde{B}}_{hi}^{T}R & 0\\ {}* & * & * & -\gamma^{2}I & 0 & \tau_{m}{\tilde{E}}_{h1i}^{T}R & 0\\ {}* & * & * & * & -\tau_{m}R & 0 & 0\\ {}* & * & * & * & * & -\tau_{m}R & 0\\ {}* & * & * & * & * & * & -I \end{array}\right]$$

$${\tilde{\Gamma }}_{3}={\tilde{\varsigma }}_{0}\Delta {\tilde{\varsigma }}_{2}+{\tilde{\varsigma }}_{2}^{T}\Delta^{T}{\tilde{\varsigma }}_{0}^{T}+{\tilde{\varsigma }}_{1}\Delta {\tilde{\varsigma }}_{3}+{\tilde{\varsigma }}_{3}^{T}\Delta^{T}{\tilde{\varsigma }}_{1}^{T}+{\tilde{\varsigma }}_{1}\Delta {\tilde{\varsigma }}_{4}+{\tilde{\varsigma }}_{4}^{T}\Delta^{T}{\tilde{\varsigma }}_{1}^{T}$$

in which

$${\tilde{\varsigma }}_{0}={\left[\begin{array}{ccccccc} 0 & Z_{2i}{ϒ}_{1} & 0 & 0 & 0 & 0 & 0 \end{array}\right]}^{T},$$

$${\tilde{\varsigma }}_{1}={\left[\begin{array}{ccccccc} 0 & {ϒ}_{1}{}^{T}Z_{2i}^{T} & 0 & 0 & 0 & 0 & 0 \end{array}\right]}^{T},{\tilde{\varsigma }}_{2}=\left[\begin{array}{ccccccc} {ϒ}_{2}{\tilde{C}}_{hi} & 0 & 0 & 0 & 0 & 0 & 0 \end{array}\right],$$

$${\tilde{\varsigma }}_{3}=\left[\begin{array}{ccccccc} 0 & 0 & {ϒ}_{2}{\tilde{D}}_{hi} & 0 & 0 & 0 & 0 \end{array}\right],{\tilde{\varsigma }}_{4}=\left[\begin{array}{ccccccc} 0 & 0 & 0 & {ϒ}_{2}{\tilde{E}}_{h2i} & 0 & 0 & 0 \end{array}\right].$$

By Lemma 1, there is

(29) $${\tilde{\Gamma }}_{3}\leq \varepsilon_{1}{\tilde{\varsigma }}_{0}{\tilde{\varsigma }}_{0}^{T}+\varepsilon_{1}{}^{-1}{\tilde{\varsigma }}_{2}^{T}{\tilde{\varsigma }}_{2}+\varepsilon_{2}{\tilde{\varsigma }}_{1}{\tilde{\varsigma }}_{1}^{T}+\varepsilon_{2}{}^{-1}{\tilde{\varsigma }}_{3}^{T}{\tilde{\varsigma }}_{3}+\varepsilon_{3}{\tilde{\varsigma }}_{1}{\tilde{\varsigma }}_{1}^{T}+\varepsilon_{3}{}^{-1}{\tilde{\varsigma }}_{4}^{T}{\tilde{\varsigma }}_{4}$$

Then by the Schur complement, $\tilde{\Gamma }<0$ is equivalent to

(30) $$\left[\begin{array}{ccccccc} {\tilde{\Gamma }}_{2} & {\tilde{\varsigma }}_{0} & {\tilde{\varsigma }}_{1} & {\tilde{\varsigma }}_{1} & {\tilde{\varsigma }}_{2}^{T} & {\tilde{\varsigma }}_{3}^{T} & {\tilde{\varsigma }}_{4}^{T}\\ {}* & -\varepsilon_{1}I & 0 & 0 & 0 & 0 & 0\\ {}* & * & -\varepsilon_{2}I & 0 & 0 & 0 & 0\\ {}* & * & * & -\varepsilon_{3}I & 0 & 0 & 0\\ {}* & * & * & * & -\varepsilon_{1}{}^{-1}I & 0 & 0\\ {}* & * & * & * & * & -\varepsilon_{2}{}^{-1}I & 0\\ {}* & * & * & * & * & * & -\varepsilon_{3}{}^{-1}I \end{array}\right]<0$$

For time-varying uncertainties of the filtering error system, based on Equations (4), (9) and Lemma 1, Equation (30) can be rewritten as

(31) $$\begin{array}{cc} \tilde{\Gamma }= & \sum_{i=1}^{r}\sum_{j=1}^{r}h_{i}\left(s\left(t\right)\right)h_{j}\left(s\left(t\right)\right)\{\Gamma_{ij}+\Psi_{1ij}K_{i}\Xi_{1i}+\Xi_{1i}^{T}K_{i}^{T}\Psi_{1ij}^{T}+\Psi_{2i}K_{i}\Xi_{2i}+\Xi_{2i}^{T}K_{i}^{T}\Psi_{2i}^{T}\\ & +\Psi_{3i}K_{a}\Xi_{3i}+\Xi_{3i}^{T}K_{a}^{T}\Psi_{3i}^{T}+\Psi_{4i}K_{l}\Xi_{4i}+\Xi_{4i}^{T}K_{l}^{T}\Psi_{4i}^{T}\}\\ \leq & \sum_{i=1}^{r}\sum_{j=1}^{r}h_{i}\left(s\left(t\right)\right)h_{j}\left(s\left(t\right)\right)\{\Gamma_{ij}+\frac{1}{\mu_{1}}\Psi_{1ij}\Psi_{1ij}^{T}+\mu_{1}\Xi_{1i}^{T}\Xi_{1i}+\frac{1}{\mu_{2}}\Psi_{2i}\Psi_{2i}^{T}+\mu_{2}\Xi_{2i}^{T}\Xi_{2i}\\ & +\frac{1}{\mu_{3}}\Psi_{3i}\Psi_{3i}^{T}+\mu_{3}\Xi_{3i}^{T}\Xi_{3i}+\frac{1}{\mu_{4}}\Psi_{4i}\Psi_{4i}^{T}+\mu_{4}\Xi_{4i}^{T}\Xi_{4i}\}<0 \end{array}$$

By applying the Schur complement to Equations (22) and (23), there are

(32) $$\begin{array}{l} \Gamma_{ii}-\Lambda_{i}+\frac{1}{\mu_{1ii}}\Psi_{1ii}\Psi_{1ii}^{T}+\mu_{1ii}\Xi_{1i}^{T}\Xi_{1i}+\frac{1}{\mu_{2i}}\Psi_{2i}\Psi_{2i}^{T}+\mu_{2i}\Xi_{2i}^{T}\Xi_{2i}+\frac{1}{\mu_{3i}}\Psi_{3i}\Psi_{3i}^{T}+\mu_{3i}\Xi_{3i}^{T}\Xi_{3i}\\ +\frac{1}{\mu_{4i}}\Psi_{4i}\Psi_{4i}^{T}+\mu_{4i}\Xi_{4i}^{T}\Xi_{4i}<0,(1\leq i\leq r) \end{array}$$

and

(33) $$\begin{array}{l} \Pi_{ij}+\frac{1}{\mu_{1ji}}\Psi_{1ji}\Psi_{1ji}^{T}+\mu_{1ij}\Xi_{1i}^{T}\Xi_{1i}+\frac{1}{\mu_{2j}}\Psi_{2j}\Psi_{2j}^{T}+\mu_{2i}\Xi_{2i}^{T}\Xi_{2i}+\frac{1}{\mu_{3j}}\Psi_{3j}\Psi_{3j}^{T}+\mu_{3i}\Xi_{3i}^{T}\Xi_{3i}\\ +\frac{1}{\mu_{4j}}\Psi_{4j}\Psi_{4j}^{T}+\mu_{4i}\Xi_{4i}^{T}\Xi_{4i}<0,(1\leq i\leq j\leq r) \end{array}$$

Then based on Equations (31)–(33), there is

(34) $$\begin{array}{ll} \tilde{\Gamma } & <\sum_{i=1}^{r}\sum_{i=1}^{r}h_{i}\left(s\left(t\right)\right)h_{i}\left(s\left(t\right)\right)\Lambda_{i}+\sum_{i=1}^{r}\sum_{j=1}^{r}h_{i}\left(s\left(t\right)\right)h_{j}\left(s\left(t\right)\right)\left[\rho_{ij}+\rho_{ij}{}^{T}\right]\\ & ={\left[\begin{array}{c} h_{1}\left(s\left(t\right)\right)\\ h_{2}\left(s\left(t\right)\right)\\ \vdots \\ h_{r}\left(s\left(t\right)\right) \end{array}\right]}^{T}\left[\begin{array}{cccc} \Lambda_{1} & \rho_{12} & \cdots & \rho_{1r}\\ {}* & \Lambda_{2} & \cdots & \rho_{2r}\\ \vdots & \vdots & \ddots & \vdots \\ {}* & * & \cdots & \Lambda_{r} \end{array}\right]\left[\begin{array}{c} h_{1}\left(s\left(t\right)\right)\\ h_{2}\left(s\left(t\right)\right)\\ \vdots \\ h_{r}\left(s\left(t\right)\right) \end{array}\right]<0 \end{array}$$

which is the same as Equation (21). The proof is completed. □

**Remark 2.**  *The fault-tolerant filter designed by Theorem 2 has robustness against any sensor failures with gain matrix* $\Theta_{i}\in \left[\Theta_{\min},\Theta_{\max}\right]$ *, where* $\Theta_{\min}$ *and* $\Theta_{\max}$ *are composed of the minimum and maximum allowable value of sensors failures, respectively. Obviously, the sensor failures are not limited to a given finite interval and converted into a dynamic interval, which expands the range of the allowed sensor failure and improves the reliability of the system.*

## 4. Simulation Example

In this section, a simulation example is given to illustrate the effectiveness of the proposed method in this paper. The system parameters are defined as follows:

$$A_{1}=\left[\begin{array}{cc} -2.63 & 0.13\\ 1.25 & -2.50 \end{array}\right],A_{2}=\left[\begin{array}{cc} -2.38 & 0\\ -0.25 & -1.38 \end{array}\right],B_{1}=\left[\begin{array}{cc} -1.1 & 0.1\\ -0.8 & -0.9 \end{array}\right],B_{2}=\left[\begin{array}{cc} -0.9 & 0\\ -1.1 & -1.2 \end{array}\right],L_{1}=\left[\begin{array}{c} -0.11\\ -0.3 \end{array}\right],$$

$$L_{2}=\left[\begin{array}{c} -0.4\\ 0.32 \end{array}\right],E_{11}=\left[\begin{array}{c} -0.5\\ 1.0 \end{array}\right],E_{12}=\left[\begin{array}{c} -0.5\\ 1.0 \end{array}\right],C_{1}=\left[\begin{array}{cc} -0.2 & 0.1\\ 0 & 0.05 \end{array}\right],C_{2}=\left[\begin{array}{cc} 0.3 & 1.0\\ 0.1 & -0.5 \end{array}\right],D_{1}=\left[\begin{array}{cc} 0.5 & 1.0\\ 0.2 & -0.3 \end{array}\right],$$

$$D_{2}=\left[\begin{array}{cc} 1.0 & -0.2\\ 0.2 & -0.5 \end{array}\right],M_{1}=\left[\begin{array}{c} 0.1\\ -0.2 \end{array}\right],M_{2}=\left[\begin{array}{c} 0.3\\ -0.7 \end{array}\right],E_{21}=\left[\begin{array}{c} 0.1\\ -0.1 \end{array}\right],E_{22}=\left[\begin{array}{c} 0.2\\ 0.3 \end{array}\right],F_{1}=\left[\begin{array}{cc} 1.0 & -0.5\\ 0.2 & -0.3 \end{array}\right],$$

$$F_{2}=\left[\begin{array}{cc} -0.2 & 0.3\\ 0.1 & 0 \end{array}\right],G_{1}=\left[\begin{array}{cc} 0.05 & 0.12\\ 0.08 & 0.06 \end{array}\right],G_{2}=\left[\begin{array}{cc} 0.01 & 0.07\\ 0.13 & 0.05 \end{array}\right],N_{1}=\left[\begin{array}{c} 0.2\\ -0.5 \end{array}\right],N_{2}=\left[\begin{array}{c} 0.7\\ -0.3 \end{array}\right],$$

$$T_{11}={\left[\begin{array}{ccc} 0 & -0.5 & 0.1 \end{array}\right]}^{T},T_{12}={\left[\begin{array}{ccc} 0 & 0.3 & 0 \end{array}\right]}^{T},T_{21}={\left[\begin{array}{cc} 0.8 & 0.1 \end{array}\right]}^{T},T_{22}={\left[\begin{array}{cc} -0.3 & 0.6 \end{array}\right]}^{T},$$

$$T_{41}={\left[\begin{array}{ccc} -0.5 & 0.1 & 0 \end{array}\right]}^{T},T_{42}={\left[\begin{array}{ccc} 0.5 & 1.0 & 0 \end{array}\right]}^{T},T_{51}={\left[\begin{array}{cc} 0.5 & 0.1 \end{array}\right]}^{T},T_{52}={\left[\begin{array}{cc} -0.2 & 0.1 \end{array}\right]}^{T},$$

$$V_{11}=\left[\begin{array}{ccc} 0 & 0.3. & 0.2 \end{array}\right],V_{12}=\left[\begin{array}{ccc} 0.5 & 0 & 0.1 \end{array}\right],V_{21}=\left[\begin{array}{ccc} 0.2 & 0 & 0 \end{array}\right],V_{22}=\left[\begin{array}{ccc} 0 & -0.2 & 0.1 \end{array}\right],V_{31}=0.1,$$

$$V_{32}=0.2\;,V_{41}=\left[\begin{array}{ccc} 0 & -0.4 & 0 \end{array}\right],V_{42}=\left[\begin{array}{ccc} 0 & -0.4 & 0.1 \end{array}\right],V_{51}=\left[\begin{array}{ccc} 0.1 & 0 & 0 \end{array}\right],V_{52}=\left[\begin{array}{ccc} 0 & -0.4 & 0 \end{array}\right].$$

Furthermore, the membership functions are

$$h_{1}=\sin^{2}\left(x_{1}\left(t\right)\right),h_{2}=\cos^{2}\left(x_{1}\left(t\right)\right)$$

The state space of time delay is τ(t)∈{1,2,3}, and the state transition matrix of τ(t) is

$$\Pi =\left[\begin{array}{ccc} -0.6 & 0.4 & 0.2\\ 0.3 & -0.5 & 0.2\\ 0.1 & 0.4 & -0.5 \end{array}\right]$$

**Figure 2:**
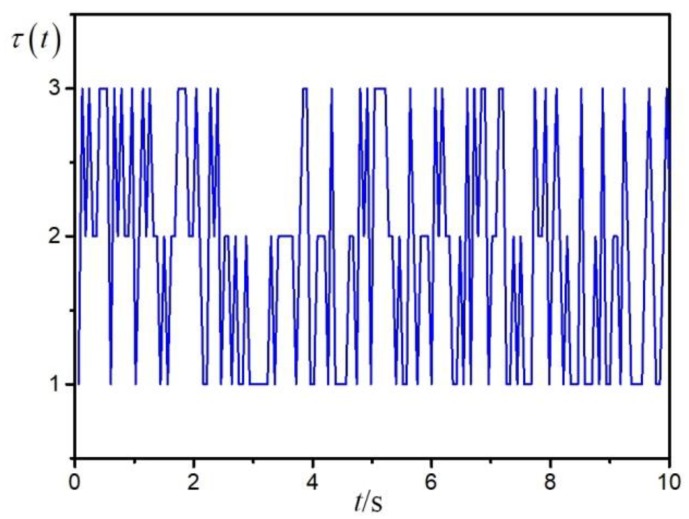
Distribution of time-delay.

Based on the matrix Π, the distribution of the Markov chain-type time-delay is shown in Figure 2. The range of sensor failures is assumed as $0\leq \alpha_{1}\leq 2.3,0.6\leq \alpha_{2}\leq 3.2$. The given value of γ is γ=2 and then the filter parameters can be solved by Theorem 2:

$$\begin{array}{l} A_{f1}=\left[\begin{array}{ccc} -6.8211 & -2.0551 & 1.3048\\ 5.6376 & -0.4909 & -2.9358\\ 2.3633 & 4.0141 & 0.5280 \end{array}\right],A_{f2}=\left[\begin{array}{ccc} -0.7015 & 1.1047 & -0.1180\\ -5.0462 & -2.8418 & 0.7740\\ -1.6330 & -2.1774 & -0.1382 \end{array}\right],\\ B_{f1}=\left[\begin{array}{cc} 4.1530 & 9.1544\\ -6.3608 & -16.3615\\ -4.7214 & -4.7216 \end{array}\right],B_{f2}=\left[\begin{array}{cc} -1.8895 & -0.4070\\ 2.4513 & 1.6992\\ 1.4846 & -0.9897 \end{array}\right],\\ L_{f1}=\left[\begin{array}{ccc} 1.0501 & -0.3799 & 0.2000\\ 0.2800 & -0.2400 & -0.5000 \end{array}\right],L_{f2}=\left[\begin{array}{ccc} -0.1908 & 0.3698 & 0.7000\\ 0.2304 & 0.0501 & -0.3000 \end{array}\right]. \end{array}$$

The system sampling period is set as T=20ms. The initial condition is defined as $x\left(0\right)={\left[\begin{array}{cc} -0.8 & 1.6 \end{array}\right]}^{T}$ and the external disturbance w(t) is

$$w\left(t\right)=\frac{3\sin\left(0.8t\right)}{0.55t^{2}+1}$$

Furthermore, the health parameter is defined as follows to represent the performance degradation during the simulation:

$$h\left(t\right)=\left\{ \begin{array}{ll} -0.02t & t<5s\\ -0.1 & t\geq 5s \end{array}\right.$$

.

**Figure 3:**
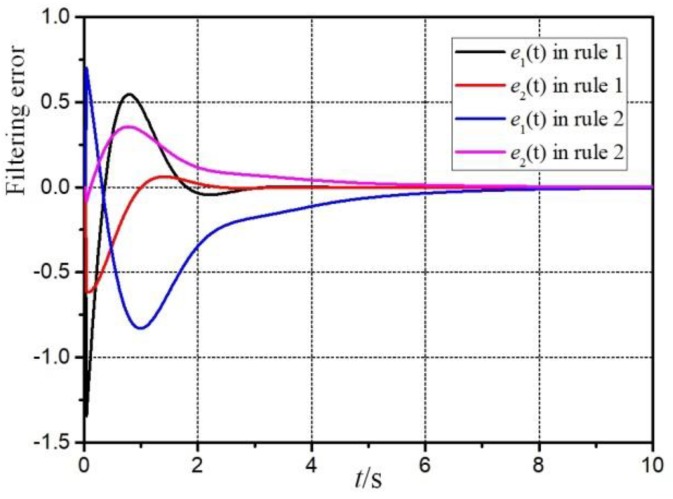
Filtering error signal e(t).

**Figure 4:**
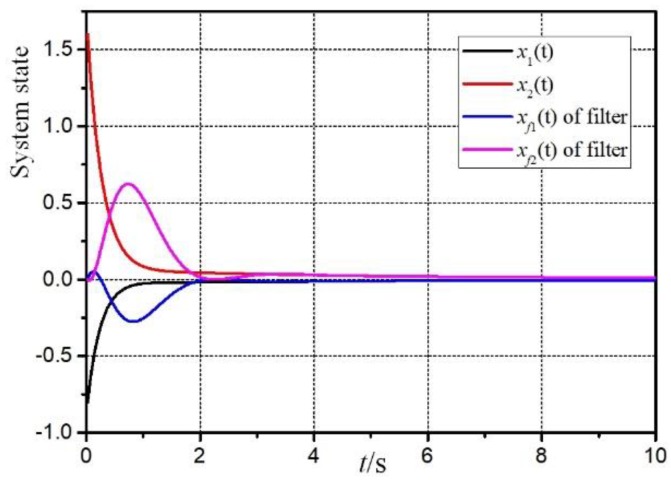
Responses of system and filter states in rule 1.

**Figure 5:**
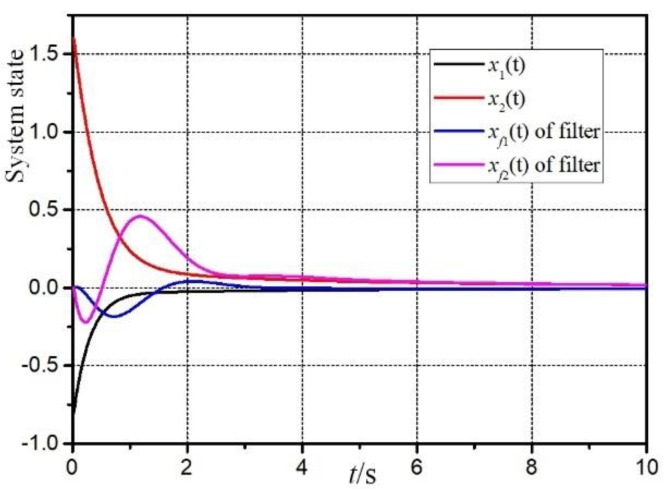
Responses of system and filter states in rule 2.

Figure 3 is the filtering error response $e\left(t\right)=z(t)-\hat{z}\left(t\right)$. It can be seen that the filter designed in this paper can remain stable under the influence of sensor failures, parameter uncertainties and external disturbance. And Figure 4 and Figure 5 are the responses of the system and filter state.

To inspect the conservativeness of the sufficient condition proposed in this paper, the minimum γ obtained by the method in this paper (defined as $\gamma_{1}$) is compared with the results solved by the method in [28] (defined as $\gamma_{2}$), as shown in Table 1. Note that, in [28] different δ values may lead to a different minimum $\gamma_{2}$, and δ has no influence on $\gamma_{1}$. It can be seen that the non-fragile filter sacrifices some conservativeness to obtain robustness against perturbation of filter parameters and sensor failures. However, from the comparison results, $\gamma_{1}$ is close to the minimum $\gamma_{2}$, which indicates that the method in this paper involves fewer sacrifices regarding conservativeness.

## 5. Conclusions

For filtering problems, sensor failures have a catastrophic effect on the filtering results. In this paper, a fault-tolerant robust non-fragile *H*∞ filter for NCSs with sensor failures is designed. First, the T-S fuzzy model for NCSs with sensor failures is constructed and the health parameters are also considered in the model to represent performance degradation. Then a desired filter is designed and the sufficient condition for the existence of the designed filter is derived in terms of LMIs solutions. In addition, a larger dynamic failure interval is used to expand the range of the allowed sensor failure. Finally, the results of the simulation show that the designed filter has robustness against sensor failures, parameter uncertainties, and external disturbance, and the method proposed in this paper has less conservativeness.

## Figures and Tables

**Table 1:** Comparison of minimum γ.

	*δ* = 0.7	*δ* = 1	*δ* = 2	*δ* = 5	*δ* = 10	*δ* = 20
*γ*_1_	1.16					
*γ*_1_	4.23	2.87	1.23	0.97	2.05	3.17
